# Street Cleaning Trucks as Potential Sources of *Legionella pneumophila*

**DOI:** 10.3201/eid2311.161390

**Published:** 2017-11

**Authors:** Natalia Valero, Mercè de Simón, Pau Gallés, Neus Izquierdo, Jaume Arimon, Raquel González, Sandra Manzanares-Laya, Ingrid Avellanes, Anna Gómez

**Affiliations:** Agència de Salut Pública de Barcelona, Barcelona, Spain (N. Valero, M. de Simón, P. Gallés, N. Izquierdo, J. Arimon, R. González, S. Manzanares-Laya, I. Avellanes, A. Gómez);; Centro de Investigación Biomédica en Red de Epidemiología y Salud Pública (CIBERESP), Barcelona (S. Manzanares-Laya)

**Keywords:** *Legionella*, environmental exposure, Legionnaires’ disease, occupational health, molecular typing, street cleaner, foam, truck, *Legionella pneumophila*, pneumonia, bacteria, Barcelona, Spain

## Abstract

In 2015, Legionnaires’ disease was diagnosed in a street cleaning worker. We found *Legionella pneumophila* serogroup 1 in the water and internal foam from the tanks of 2 trucks used by the worker during the incubation period. The internal foam was removed, and a *Legionella* prevention program was implemented.

Legionnaires’ disease (LD) is an acute pneumonia caused by inhalation or aspiration of aerosols contaminated with *Legionella* bacteria. *Legionella* spp. are ubiquitous in freshwater aquatic habitats. Multiplication of *Legionella* in artificial water systems is facilitated by temperatures around 35°C and factors such as lack of disinfection, water stagnation, and poor maintenance (*1*).

In Spain, 925 cases of LD were reported in 2014, of which 82% were sporadic cases, not related to outbreaks (*2*). LD outbreaks have been related to cooling towers, spa pools, and water distribution systems (*2**–**4*), which are considered by regulations in Spain to have a high probability of proliferation of *Legionella* (*5*).

The identification of exposure in sporadic cases provides a good opportunity to enhance understanding of reservoirs for *Legionella* in relatively rare sources (*6**,**7*). This case report summarizes the environmental study conducted to identify the possible source of exposure of a street cleaning worker who contracted LD.

## The Study

A 58-year-old man in Barcelona who smoked began to show symptoms compatible with legionellosis on July 27, 2015. On August 11, LD was diagnosed in the patient; this diagnosis was confirmed by a urinary antigen test (UAT). On August 14, the case was reported to the Public Health Agency of Barcelona.

A trained public health practitioner interviewed the affected patient by using a standardized questionnaire to obtain information on demographic data, personal risk factors, and activities during the 15 days before the onset of illness. The only exposure to sources of aerosolized water highlighted by the epidemiologic survey was an occupational exposure to high-pressure water hoses from street cleaning trucks during the incubation period. The trucks used had 2-m^3^ water tanks with an internal foam lining ≈15 cm thick. The purpose of the foam was to help steady the truck when in motion. The truck tanks were filled with groundwater untreated with chlorine or with drinking water from the town’s water supply network, depending on their availability during the journey. At the end of the day, the tanks were emptied but the internal foam remained impregnated with water. Once a year, the tanks were disinfected with a 20-ppm chlorine solution for 2 hours; hoses were replaced relatively often because of deterioration. However, the interior walls of the tanks were never cleaned, and the foam lining was never replaced.

Three days after the interview, we took samples from the 4 trucks that the worker used in the 15 days before symptom onset. We took water samples and smears from the hoses and water tanks to test them for *Legionella*. We also took a water sample from the groundwater tank where the trucks were usually filled.

We analyzed the samples in accordance with the ISO 11731 method for counting *Legionella* spp. (*8*). In addition, we obtained counts of *L. pneumophila* serogroup 1 and of serogroups 2–15. We performed molecular typing of the strains of *L. pneumophila* serogroup 1 isolated in the different samples via DNA macrorestriction and subsequent pulsed field electrophoresis. We calculated the similarity coefficient and displayed the results of molecular typing as a dendrogram generated by the FPQuest software package (Bio-Rad Laboratories, Hercules, CA, USA).

Water sample temperatures ranged from 26°C to 28°C. We detected *L. pneumophila* serogroup 1 in the water from 2 of the 4 trucks sampled. *L. pneumophila* serogroup 1 counts were 150 CFU/L for truck 1 and 1,000 CFU/L for truck 2 (Table). We did not detect *Legionella* in the sample of the groundwater tank or in the hose smears of either truck.

**Table:** Counts of *Legionella pneumophila* serogroups 1 and 2–15 in the samples of water, foam, and smears analyzed before and after cleaning and disinfection of 2 street cleaning trucks, Barcelona, Spain, 2015*

Location and sample type	Before cleaning and disinfection, CFU/L			After cleaning and disinfection, CFU/L	
	*L. pneumophila* serogroup 1	*L. pneumophila* serogroups 2–15		*L. pneumophila* serogroup 1	*L. pneumophila* serogroups 2–15
Truck 1					
Water tank	150	<1†		400	<1
Hose water	150	<1		5,700	<1
Foam sample 1	NA	NA		250‡	Not detected
Foam sample 2	NA	NA		5,500‡	Not detected
Tank surface smear	NA	NA		Detected	Not detected
Truck 2					
Water tank	1,000	1,000		125	125
Hose water	<1	50		<1	50
Foam sample 1	NA	NA		275‡	125‡
Foam sample 2	NA	NA		10‡	10‡
Foam sample 3	NA	NA		Not detected	10‡
Foam sample 4	NA	NA		10‡	Not detected
Tank surface smear	NA	NA		Not detected	Not detected

*NA, not analyzed. †Limit of detection in water samples: 1 CFU/L. ‡Counts for 250 cm^3^ of foam.

After *Legionella* detection, the tanks were disinfected and cleaned following the cleaning procedure described by law for hot water systems (*5*). After 15 days, we drew new water samples to assess the effectiveness of the disinfection, and after 23 days, we took samples of the foam and from the internal surfaces of the tanks of both trucks. We obtained samples of foam by cutting out pieces (4 × 4 × 15 mm) with a sterile scalpel and placed them in sterile containers with Ringer’s solution for subsequent *Legionella* analysis. The results indicated the presence of *L. pneumophila* serogroup 1 in the foam and water tanks of both trucks and in the hose water sample and the smear from the tank of truck 1 (Table).

The results of molecular typing indicated that clones were detected in truck 1 (Dice similarity coefficient 100%) belonging to the same strain found in the hose water, in the tank water, and in the foam. We also detected another clone, genetically related to the first (Dice similarity coefficient 89%), in the tank water, in the smears of the surface of the tank, and in the foam. We identified 2 different molecular patterns in the strains isolated in the tank water and in the foam in truck 2; both patterns appeared in both tank water and foam (Figure). Regarding the foam, we obtained similar results in the foam underneath decorative stones in an ornamental fountain that caused an LD outbreak in a hospital (*9*).

**Figure:**
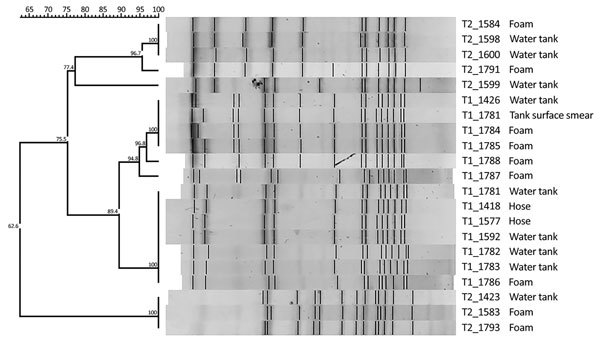
Dendrogram generated by FPQuest software showing the pattern of SfiI bands for isolates of *Legionella pneumophila* from 2 street cleaning trucks, Barcelona, Spain, 2015*.* Strains are identified with code of truck of origin (T1, T2) and an internal number. Scale bar represents Dice similarity coefficient percentage.

Weather conditions were warm in July 2015, with an average temperature >25°C even at night (*10*). These temperatures, along with water stagnation, would have favored proliferation of the bacteria in the water tanks.

Although the mode of transmission is not clear, the high-pressure hose used by the worker probably discharged aerosols containing bacteria that could be inhaled (*7*). The worker did not use a protective facemask and was the second employee in the same company who was working with cleaning trucks to contract LD. Four years earlier, another case of LD had occurred in similar conditions (J. Cayla, Public Health Agency of Barcelona, pers. comm., 2011 July 22).

Published criteria (*11*) indicate that cleaning trucks are a potential source of *Legionella*. The level of evidence of this study corresponds to level III, because *L. pneumophila* subgroup 1 was isolated in the trucks used by the worker but clinical and environmental strains could not be compared because he was diagnosed by a UAT. Furthermore, because UAT detects only *L. pneumophila* serogroup 1, lack of respiratory culture can lead to missed diagnoses of other species and serogroups; fortunately, this limitation does not apply here.

A report describing similar exposure concerned an asphalt paving machine causing an outbreak of *Legionella* in 2009 (*6*). Like the trucks in this study, this was a mobile source, and the use of untreated water had also contributed to tank pollution. Although some studies have associated LD with occupations such as gardening (*12**,**13*) and professional driving (*14*), no studies have related LD to street cleaning.

## Conclusions

We detected *L. pneumophila* serogroup 1 in 2 of the 4 trucks used by the affected worker during the incubation period; either truck could have been the source of exposure. The results of this study show that the internal foam of the tanks could act as a reservoir for *L. pneumophila* and that maintenance and routine cleaning (without removing the foam) did not prevent *Legionella* proliferation.

After the occurrence of this case, the internal foam in the truck tanks was removed, and the water tanks were cleaned again. The company responsible for the street cleaning trucks adopted a water management plan, with the agreement of the Public Health Agency of Barcelona, based on Hazard Analysis and Critical Control Points methodology (*15*) and stricter control measures. Workers are now required to wear personal protective equipment during work-related exposure.
